# Supplementary material for: Explaining Age at Autism Spectrum Diagnosis in Children with Migrant and Non-Migrant Background in Austria
Source: Brain Sci. 2020 Jul 14;10(7):448. doi: 10.3390/brainsci10070448 (PMC7407505; doi:10.3390/brainsci10070448)
Supplement: Supplementary file 1 [file brainsci-10-00448-s001.pdf]

Supplementary:

Table 1. Sample characteristics by migration status based on multiple imputed data.

			Total (N=211)	Non-migrants (n=91)	Migrants (n=120)	Difference	
	Values	%M	*M* [*SD*] or n[b] (%)	*M* [*SD*] or n[b] (%)	*M* [*SD*] or n[b] (%)	*ES r*[a]	*p*
Age at diagnosis	*12 - 119 m.o.*	0%	*46.7 [22.80]*	*53.96 [26.86]*	*41.19 [17.33]*	0.278	*<.001*
**Sociodemographic characteristics**							
Family residence in urban area	No/Yes	0%	148 (70.1%)	51 (56.0%)	97 (80.8%)	0.268	<0.001
Distance to hospital	*0 – 187 km*	0%	*38.27 [37.50]*	*49.27 [41.67]*	*29.93 [31.73]*	0.256	<0.001
Parental level of education above High school	No/Yes	41%	(28.3%)	(38.8%)	(20.3%)	0.204	0.017
**Clinical characteristics**							
Male gender	No/Yes	0%	174 (82.5%)	77 (84.6%)	97 (80.8%)	0.049	0.474
Non-verbal Developmental Quotient, (D/IQ)	*16 - 122*	13%	*62.06 [19.02]*	*65.62[21.16]*	*59.36 [16.69]*	0.136	0.026
D/IQ 50 or below	No/Yes	13%	(23.7%)	(22.3%)	(24.7%)	0.028	0.701
Expressive Language Quotient (ELQ)	*13 -128*	15%	*48.02 [25.42]*	*58.24 [29.03]*	*40.27 [18.89]*	0.350	*<0.001*
ELQ 50 or below	No/Yes	15%	(66.3%)	(49.0%)	(79.3%)	0.318	*<0.001*
Receptive Language Quotient (RLQ)	*15 - 133*	15%	*49.62 [25.51]*	*60.46 [29.08]*	*41.39 [18.58]*	0.370	*<0.001*
RLQ score 50 or below	No/Yes	15%	(63.6%)	(46.2%)	(76.8%)	0.315	*<0.001*
ADOS-Calibrated Severity Scores -Total CSS							
Social Affect-CSS	1 - 10	18%	*6.35 [2.25]*	*5.85 [2.25]*	*6.72 [2.18]*	0.191	0.011
Repetitive Behaviour-CSS	1 - 10	18%	*5.86 [1.92]*	*5.84 [1.93]*	*5.87 [1.90]*	0.009	0.907
ICD-10 ASD Code							
Autism disorder		0%	122 (57.8%)	38 (41.8%)	84 (70.0%)	0.385	<0.001

Asperger disorder			21 (10.0%)	20 (22%)	1 (0.8%)		
PDD-nos			68 (32.2%)	33(36.3%)	35 (29.2%)		
**Referred by Pediatrician**	No/Yes	27%	(49.7%)	(35.8%)	(60.2%)	0.242	<0.001

Note. %M = percentage missing values, M = mean, SD = standard deviation. Data for variables with missing values are based on multiply imputed data. [a] Effect size estimates for differences between migrants and non-migrants in a correlation metric. [b] Due to possible decimal case numbers, no n is reported for imputed categorical variables.

Table 2. Regression models including interactions for age at diagnosis.

	**M1**	**M2**	**M3**	**M4**	**M5**	**M6**	**M7**	**M8**	**M9**
	**b (SE)**	**b (SE)**	**b (SE)**	**b (SE)**	**b (SE)**	**b (SE)**	**b (SE)**	**b (SE)**	**b (SE)**
**Sociodemografic characteristics**									
Family residence in urban area	−0.465 (10.361)	1.101 (3.480)	1.285 (3.440)	1.035 (3.429)	.985 (3.470)	1.196 (3.377)	1.156 (3.461)	1.191 (3.482)	1.143 (3.463)
Distance to hospital in Km	−0.020 (0.038)	−0.006 (0.113)	−0.027 (0.038)	−0.021 (0.038)	−0.023 (0.038)	−0.019 (0.038)	−0.020 (0.038)	−0.021 (0.038)	−0.025 (0.039)
Parental level of education above High school	−3.785 (4.102)	−3.774 (4.116)	11.575 (14.795)	−3.827 (4.092)	−4.086 (4.121)	−4.782 (4.209)	−3.698 (4.128)	−3.683 (4.051)	−3.122 (4.113)
Migration status	−6.993 (4.999)	−5.821 (4.583)	−3.856 (4.075)	5.856 (9.281)	2.300 (10.631)	1.559 (8.295)	−4.297 (9.185)	−19.013 (13.912)	−9.602* (4.546)
**Clinical characteristics**									
Male gender	1.553 (3.430)	1.538 (3.438)	1.482 (3.426)	18.058 (13.692)	1.519 (3.465)	1.940 (3.454)	1.468 (3.424)	1.210 (3.353)	1.204 (3.350)
Non-verbal Developmental Quotient	−0.209* (0.098)	−0.211* (0.099)	−0.201* (0.098)	−0.215* (0.097)	−0.003 (0.280)	−0.205* (0.099)	−0.210* (0.098)	−0.213* (0.096)	−0.213* (0.097)
Language Composite (expressive and receptive)	3.639*** (0.903)	3.645*** (0.905)	3.219*** (0.985)	3.743*** (0.907)	3.581*** (0.904)	5.868* (2.496)	3.621*** (0.911)	3.282*** (1.019)	3.707*** (0.903)
ADOS-Calibrated Severity Scores -Total CSS									
Social Affect-CSS	−0.134 (.741)	-.131 (.741)	−0.184 (0.732)	−0.172 (0.740)	−0.169 (0.738)	−0.266 (0.763)	0.347 (2.538)	−0.364 (0.788)	−0.153 (0.734)

Repetitive Behaviour-CSS	−3.568*** (0.946)	-3.571 *** (0.948)	−3.553*** (0.926)	−3.506*** (0.931)	−3.495*** (0.952)	−3.405*** (1.002)	−3.546*** (0.945)	−6.977 (3.613)	−3.572*** (0.937)
**Referred by Pediatrician**	−9.407** (3.375)	−9.346* (3.396)	−10.155** (3.473)	−9.059** (3.353)	−9.376** (3.353)	−9.665** (3.337)	−9.363** (3.374)	−9.499** (3.342)	−21.697 (12.124)
**Interactions**									
Migration status × Family Residence in Urban Area	1.132 (6.089)								
Migration status × Home Distance to hospital		−0.010 (0.063)							
Migration status × Parental Level of Education			−9.948 (8.375)						
Migration status × Male Gender				−10.234 (7.428)					
Migration status × Non-verbal Developmental Quotient					−.139 (0.158)				
Migration status × Language Composite						−1.627 (1.604)			
Migration status × Social Affect							−0.311 (1.444)		
Migration status × Repetitive Behaviour								2.110 (2.139)	
Migration status × Referred by Pediatrician									7.733 (6.751)
R^2	0.346	0.346	0.355	0.353	0.350	0.351	0.347	0.354	0.353

Note. Results are based on multiple imputed data. *** p<.001, ** p<.01, * p<.05.

Table 3. Languages reported as the primary language in the families with migrant background grouped by geographical areas (*n* = 120).

**Southeastern/Eastern Europe** *n* **= 65**	**Western/Northern Europa** *n* **= 42**	**Middle East** *n* **= 7**	**Asia** *n* **= 4**	**Others** *n* **= 2**
Albanian	English	Arabic	Chinese	Ethiopian
Bosnian	Italian	Farsi	Mongolian	
Bulgarian	Dutch	Turkish<br>Kumykish	Bengali	
Lithuanian	Portuguese	Persian	Pandschabi	
Macedonian	Spanish			
Rumanian				
Serbo-Croatian				
Chechen				
Slovakian				
Hungarian				
